# Involvement of Protein Kinase R in Double-Stranded RNA-Induced Proteasomal Degradation of Hypoxia Inducible Factor-1α

**DOI:** 10.1007/s10753-023-01881-8

**Published:** 2023-08-24

**Authors:** Takuma Hotani, Kanako Nakagawa, Tomohito Tsukamoto, Hiroyuki Mizuguchi, Fuminori Sakurai

**Affiliations:** 1 Laboratory of Biochemistry and Molecular Biology, Graduate School of Pharmaceutical Sciences, Osaka University, 1-6 Yamadaoka, Suita, Osaka 565-0871 Japan; 2 The Center for Advanced Medical Engineering and Informatics, Osaka University, 2-2 Yamadaoka, Suita, Osaka 565-0871 Japan; 3 Laboratory of Functional Organoid for Drug Discovery, Center for Drug Discovery Resources Research, National Institute of Biomedical Innovation, Health and Nutrition, 7-6-8 Saito, Asagi, Ibaraki, Osaka 567-0085 Japan; 4 Integrated Frontier Research for Medical Science Division, Institute for Open and Transdisciplinary Research Initiatives, Osaka University, Osaka, 565-0871 Japan; 5 Center for Infectious Disease Education and Research (CiDER), Osaka University, Osaka, 565-0871 Japan

**Keywords:** Double-stranded RNA, HIF-1α, Proteasome, Ubiquitin, PKR

## Abstract

Hypoxia inducible factor-1α (HIF-1α) is a crucial therapeutic target in various diseases, including cancer and fibrosis. We previously demonstrated that transfection with double-stranded RNA (dsRNA), including polyI:C and the dsRNA genome of mammalian orthoreovirus, resulted in significant reduction in HIF-1α protein levels in cultured cells; however, it remained to be elucidated how dsRNA induced down-regulation of HIF-1α protein levels. In this study, we examined the mechanism of dsRNA-mediated down-regulation of HIF-1α protein levels. We found that among the various cellular factors involved in dsRNA-mediated innate immunity, knockdown and knockout of protein kinase R (PKR) significantly restored HIF-1α protein levels in dsRNA-transfected cells, indicating that PKR was involved in dsRNA-mediated down-regulation of HIF-1α. Proteasome inhibitors significantly restored the HIF-1α protein levels in dsRNA-transfected cells. Ubiquitination levels of HIF-1α were increased by transfection with dsRNA. These findings indicated that degradation of HIF-1α in a ubiquitin–proteasome pathway was promoted in a PKR-dependent manner following dsRNA transfection. Expression of not only HIF-1α but also several proteins, including CDK4 and HER2, was down-regulated following dsRNA transfection. These data provide important clues for elucidation of the mechanism of dsRNA-mediated cellular toxicity, as well as for therapeutic application of dsRNA.

**Supplementary Information:**

The online version contains supplementary material available at 10.1007/s10753-023-01881-8.

## Introduction

Hypoxia inducible factor (HIF)-1α is a transcriptional factor subunit crucial for regulation of cellular adaptive responses to hypoxia. HIF-1α forms a heterodimeric complex, HIF-1α, with HIF-1α, and then binds to the hypoxia-responsive element (HRE) in HIF**-**1α target genes, leading to the transcription of HIF-1α target genes, including vascular endothelial growth factor (VEGF) and glucose transporter 1 (GLUT1). HIF-1α has been demonstrated to be involved in various diseases, including cancer, fibrosis, and ischemic heart disease [1–3]. In particular, high levels of HIF-1α expression are often found in various types of cancers because of efficient consumption of oxygen and dysfunctional vascularization in cancers. Therefore, HIF-1α is a promising therapeutic target for cancer therapy. Several HIF-1α inhibitors have been tested in clinical trials of various types of cancers [4, 5].

HIF-1α protein levels are usually regulated by oxygen levels. Under normoxic conditions, prolyl hydroxylase (PHD) and factor inhibiting HIF-1α (FIH-1) mediate hydroxylation of HIF-1α, leading to ubiquitination of HIF-1 by the Von Hippel-Lindau (VHL) protein and proteasomal degradation [6, 7]. Under hypoxic conditions, HIF-1α is stabilized through the inhibition of hydroxylation. In addition to VHL, several factors involved in regulation of HIF-1α protein levels, including RACK1 and heat shock protein (HSP) 90, have been reported[8, 9].

Several groups, including ours, demonstrated that the mammalian orthoreovirus type 3 Dearing strain (hereafter reovirus), which possesses a 10 segmented double-stranded RNA (dsRNA) genome of approximately 1–3 kb in length significantly reduced HIF-1α protein levels in cultured cells and mouse subcutaneous tumors following infection [10–13]. It is well known that reovirus is actively studied as an oncolytic virus, which replicates in a tumor cell-specific manner and efficiently kill tumor cells, and has been tested in clinical trials against various types of tumors, including phase III trials [14, 15]. Reovirus-mediated reduction in HIF-1α led to a reduction in the expression of hypoxia-responsive element-containing genes in not only cultured cells but also subcutaneous tumors [10]. These findings suggest that reovirus-mediated down-regulation of HIF-1α contributes to the antitumor effects of reovirus. We previously demonstrated that transfection with reovirus dsRNA genome and synthetic dsRNA, polyI:C, resulted in an efficient reduction in HIF-1α protein levels in cultured tumor cell lines, suggesting that among the virus components, the virus dsRNA genome played a crucial role in the reovirus-induced down-regulation of HIF-1 protein levels [11]. The sequence of the dsRNA genome was irrelevant to the reduction in HIF-1α expression; however, the mechanism of dsRNA-induced down-regulation of HIF-1α remains to be elucidated.

In this study, we examined the mechanism of dsRNA-induced down-regulation of HIF-1α. Our results showed that protein kinase R (PKR) played a crucial role in the dsRNA-induced down-regulation of HIF-1α. Proteasome inhibitors significantly restored the HIF-1α protein levels in dsRNA-transfected cells. Ubiquitination levels of HIF-1α were promoted following transfection with dsRNA. These data indicated that transfection with dsRNA resulted in activation of PKR, leading to proteasome degradation of HIF-1α.

## Materials and Methods

### Cells and Reagents

Human and mouse cultured cells were maintained in an appropriate culture medium containing 5% or 10% fetal bovine serum (FBS) and antibiotics at 37°C in a 5% CO_2_ atmosphere. For hypoxic exposure, cells were incubated in a hypoxic chamber (Veritas Co., Tokyo, Japan) in the presence of 1% O_2_ and 5% CO_2_/balance N_2_ at 37°C. MG-132, 2-aminopurine (2-AP), and PYZD4409 were purchased from Sigma (St. Louis, MO). Epoxomicin was purchased from Calbiochem (LaJolla, CA).

## Preparation of PKR-Knockout H1299 Cells

Plasmid DNAs expressing Cas9 and guide RNA against PKR, pX330-PKR-1 and pX330-PKR-2 were constructed using pX330-U6-Chimeric-BB-CBh-hSpCas9 (Addgene, #42,230) [16] and synthetic oligonucleotides. Targeted sequences of guide RNAs in the PKR gene were 5’-TCTCTTCCATTGTAGGATAC-3’ for pX330-PKR-1, and 5’-AATACATACCGTCAGAAGCA-3’ for pX330-PKR-2. Detailed information about the plasmids is available on request. H1299 cells were transfected with pX330-PKR-1, pX330-PKR-2, and a puromycin-resistant gene-expressing plasmid, pDonor-ENTR-EF1a-PuroR, using Lipofectamine 2000 (Life Technologies, Carlsbad, CA). PKR-KO H1299 cells were isolated by cultivation in the presence of puromycin. Knockout of the PKR gene was confirmed by western blotting analysis.

## Virus Purification and Virus Genome RNA Extraction

Reovirus was grown in L929 cells and purified by CsCl ultracentrifugation, followed by overnight dialysis as previously described [17]. Biological titers of reovirus were determined by a plaque-forming assay using L929 cells. The reovirus dsRNA genome was extracted from purified reovirus particles using an RNeasy Mini Kit (QIAGEN, Venlo, NL) according to the manufacturer's instructions.

## Transfection with the Reovirus dsRNA Genome

Cells were seeded at 4 × 10^4^ cells/well on a 24-well plate. On the following day, cells were transfected with the reovirus dsRNA genome at the final concentration of 4 ng/ml using Lipofectamine RNAiMAX (Life Technologies). Cells were incubated in a hypoxic chamber for 24 h, followed by western blotting analysis. When cells were treated with several inhibitors, cells were transfected with the reovirus dsRNA genome after pretreatment with the inhibitors at the following concentrations: MG-132, 10 mM; epoxomicin, 1 mM; 2-AP, 10 mM; PYZD4409, 20 mM. The cell lysates were prepared using RIPA buffer, followed by western blotting analysis as described below.

## Transfection with Small Interfering RNAs

Cells were transfected with siRNAs at a final concentration of 50 nM using Lipofectamine RNAiMAX. ON-TARGETplus siRNAs targeting human RIG-I, DDX1, DDX3, DDX60, and DHX33 were purchased from Dharmacon (Waltham, MA). An siRNA targeting human MDA5 was purchased from Invitrogen (Carlsbad, CA). siRNAs targeting the other genes were purchased from Thermo Fisher Scientific (Carlsbad, CA) and Gene Design Inc. (Osaka, Japan). The target sequences of siRNAs are shown in Supplemental Table 1. Negative control siRNA (AllStars Negative Control siRNA) was purchased from QIAGEN. Following a 48-h incubation, cells were transfected with the reovirus dsRNA genome and cultured under hypoxic conditions as described above. HIF-1α protein levels were analyzed by western blotting 24 h after reovirus dsRNA genome transfection. Knockdown efficiency was analyzed by real-time RT-PCR 48 h after siRNA transfection using primers described previously [10, 18].

## Western Blotting Analysis

Total protein was recovered from cells using RIPA buffer (Thermo Fisher Scientific) containing protease inhibitor cocktail (Sigma). The lysates were analyzed by SDS-PAGE, followed by electro-transfer onto a PVDF membrane. Western blotting analyses were carried out by using anti-HIF-1α monoclonal antibody (clone: 54/HIF-1α, 1:1000; BD Biosciences, San Jose, CA), anti-PKR monoclonal antibody (clone: FJ-6, 1:1000; Santa Cruz, Santa Cruz, CA), anti-phospho PKR monoclonal antibody (clone: E120, 1:1000; Abcam, Cambridge, MA), anti-HER2 monoclonal antibody (clone: EP1045Y, 1:1000; Abcam), anti-CDK4 monoclonal antibody (clone: D9G3E, 1:1000; Cell Signaling Technology, Boston, MA), anti-AKT monoclonal antibody (clone: C67E7, 1:1000; Cell Signaling Technology), and anti--actin monocloal antibody (clone: AC-15, 1:10,000; Sigma) as primary antibodies and Chemi-Lumi One Super (Nacalai Tesque, Kyoto, Japan). Images were captured using an LAS-4000 system (Fujifilm, Tokyo, Japan).

## Real-time RT-PCR Analysis

Total RNA was recovered from cells using ISOGEN (Nippon Gene, Tokyo, Japan) according to the manufacturer’s instructions. Complementary DNA (cDNA) was synthesized using a Superscript VILO cDNA synthesis kit (Thermo Fisher Scientific). Real-time RT-PCR was performed using THUNDERBIRD SYBR qPCR Mix (TOYOBO, Osaka, Japan) and a StepOnePlus System (Thermo Fisher Scientific). The values were normalized by the mRNA levels of a housekeeping gene, 18S ribosomal RNA (18S rRNA). The sequences of primers used in this study were described [10, 18].

## Analysis of Ubiquitination Levels of HIF-1α

H1299 cells were transfected with the reovirus dsRNA genome as described above. Total protein was recovered from cells using BlastR lysis buffer containing de-ubiquitination inhibitor and protease inhibitor cocktail (Cytoskeleton, Denver, CO). The lysates were analyzed by immunoprecipitation using a Signal-Seeker Ubiquitination Detection Kit (Cytoskeleton), followed by western blotting analysis using anti-HIF-1α antibody.

## Results

### PKR is Involved in dsRNA-Mediated Down-Regulation of HIF-1α

**Fig. 1:**
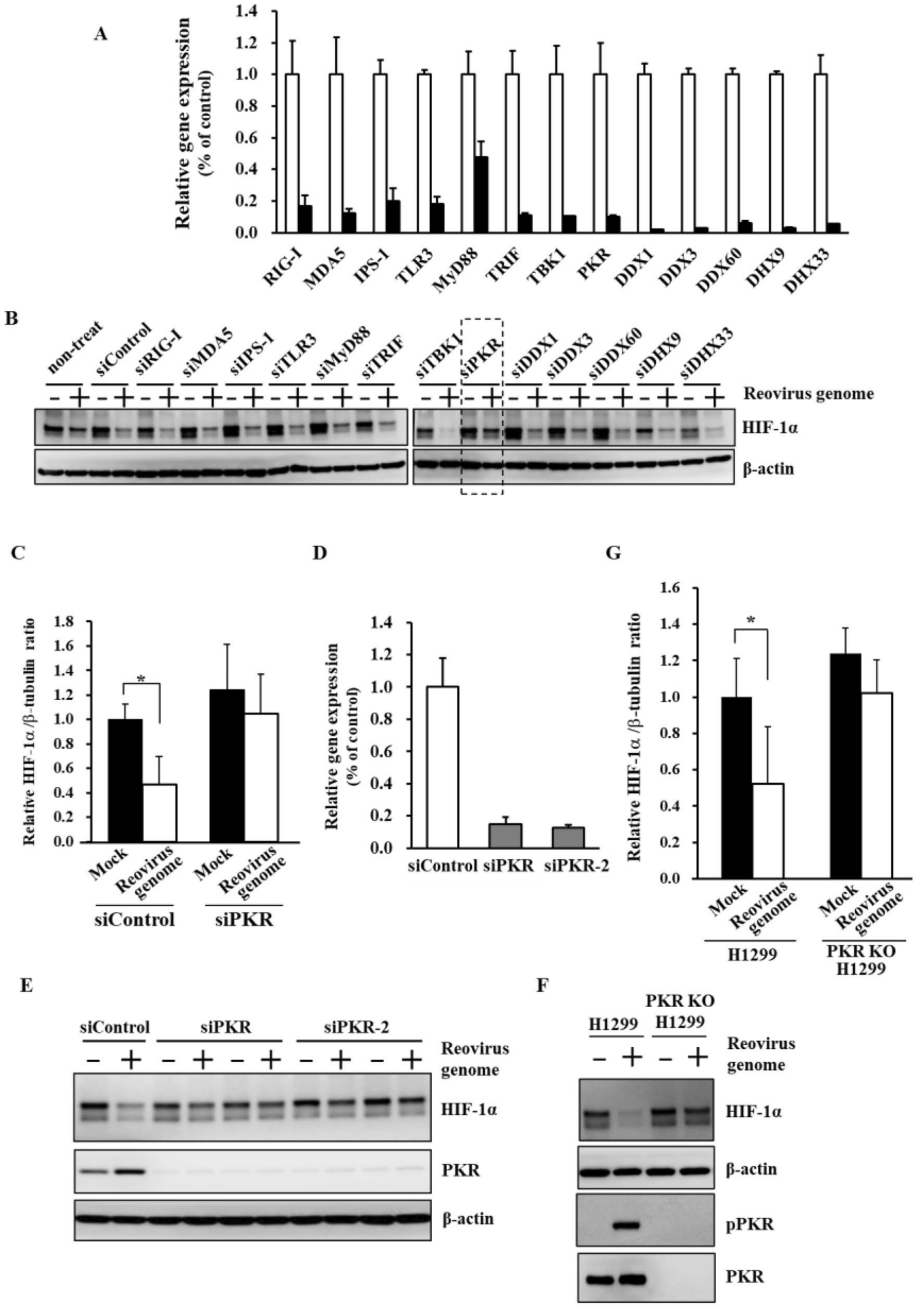
Involvement of PKR in dsRNA-mediated down-regulation of HIF-1α. **A** Knockdown efficiencies of siRNAs against dsRNA-mediated innate immunity-related genes. H1299 cells were transfected with control siRNA (open bar) and siRNAs against dsRNA-mediated innate immunity-related genes (closed bar) at 50 nM. Knockdown efficiencies were determined 24 h after transfection by real-time RT-PCR analysis. **B** HIF-1α expression levels in H1299 cells with knockdown of dsRNA-mediated innate immunity-related genes following transfection with the reovirus dsRNA genome. H1299 cells were transfected with siRNAs at 50 nM for 24 h, followed by transfection with the reovirus dsRNA genome at 4 ng/ml for 24 h. **C** quantitation of results shown in **B** and independent replicates (n = 3-4). Statistical significance relative to mock cells pretreated with control siRNA was tested by paired t test. *p < 0.05. Data are expressed as means ± S.D. (n = 3-4). **D** Knockdown efficiencies of siRNAs against PKR. H1299 cells were transfected with control siRNA and siRNAs against PKR at 50 nM. Knockdown efficiencies were determined as described above. **E** Restoration of HIF-1α expression in reovirus dsRNA genome-transfected H1299 cells by PKR knockdown using PKR-targeted siRNAs with different sequences. **F** Restoration of HIF-1α expression in reovirus dsRNA genome-transfected H1299 PKR-KO cells. H1299 PKR-KO cells were transfected with the reovirus dsRNA genome at 4 ng/ml for 24 h. **G** quantitation of results shown in **F** and independent replicates (n = 5). Statistical significance relative to mock cells was tested by paired t test. *p < 0.05. Data are expressed as means ± S.D. (n = 5). The representative images from at least three independent experiments are shown.

In order to examine which genes were involved in dsRNA-mediated down-regulation of HIF-1α, 13 genes that have been reported to be involved in dsRNA-mediated innate immunity were knocked down by siRNAs. In this study, the reovirus dsRNA genome, which consists of 1–3 kb of dsRNA and was extracted from the purified virus particles, was used as a model of virus-derived dsRNA. We found that detectable levels of a virus protein, sigma3, were not found in the cells following transfection of the reovirus dsRNA genome (Supplemental Fig. 1), suggesting that transfection of the reovirus dsRNA genome did not result in detectable levels of virus protein production. A statistically significant reduction in cell viability was not found following the dsRNA genome transfection under the experimental condition used in this study (Supplemental Fig. 2). More than 80% knockdown efficiencies were found in all the genes except for MyD88 in H1299 cells (Fig. 1A). An siRNA against MyD88 mediated approximately 60% knockdown in MyD88 mRNA levels. HIF-1α protein levels were significantly reduced following transfection with the reovirus dsRNA genome (Fig. 1B, C), as previously reported [11]. When PKR was knocked down, HIF-1α protein levels were apparently recovered in dsRNA-transfected cells. Knockdown of the other genes did not cancel the dsRNA-mediated reduction in HIF-1 protein levels. When another siRNA against PKR (siPKR-2) was used, dsRNA-induced down-regulation of HIF-1α was also canceled (Fig. 1D, E). dsRNA-induced reduction in HIF-1α protein levels was not found in PKR knockout (PKR-KO) H1299 cells (Fig. 1F, G). These results indicated that PKR played a crucial role in dsRNA-mediated down-regulation of HIF-1α. On the other hand, reovirus-mediated reduction in HIF-1α protein levels was not canceled by PKR knockdown (Supplemental Fig. 3), suggesting that reovirus down-regulated HIF-1α in a PKR-independent manner.

## Phosphorylation Activity of PKR is Indispensable for dsRNA-Mediated Down-Regulation of HIF-1α

**Fig. 2:**
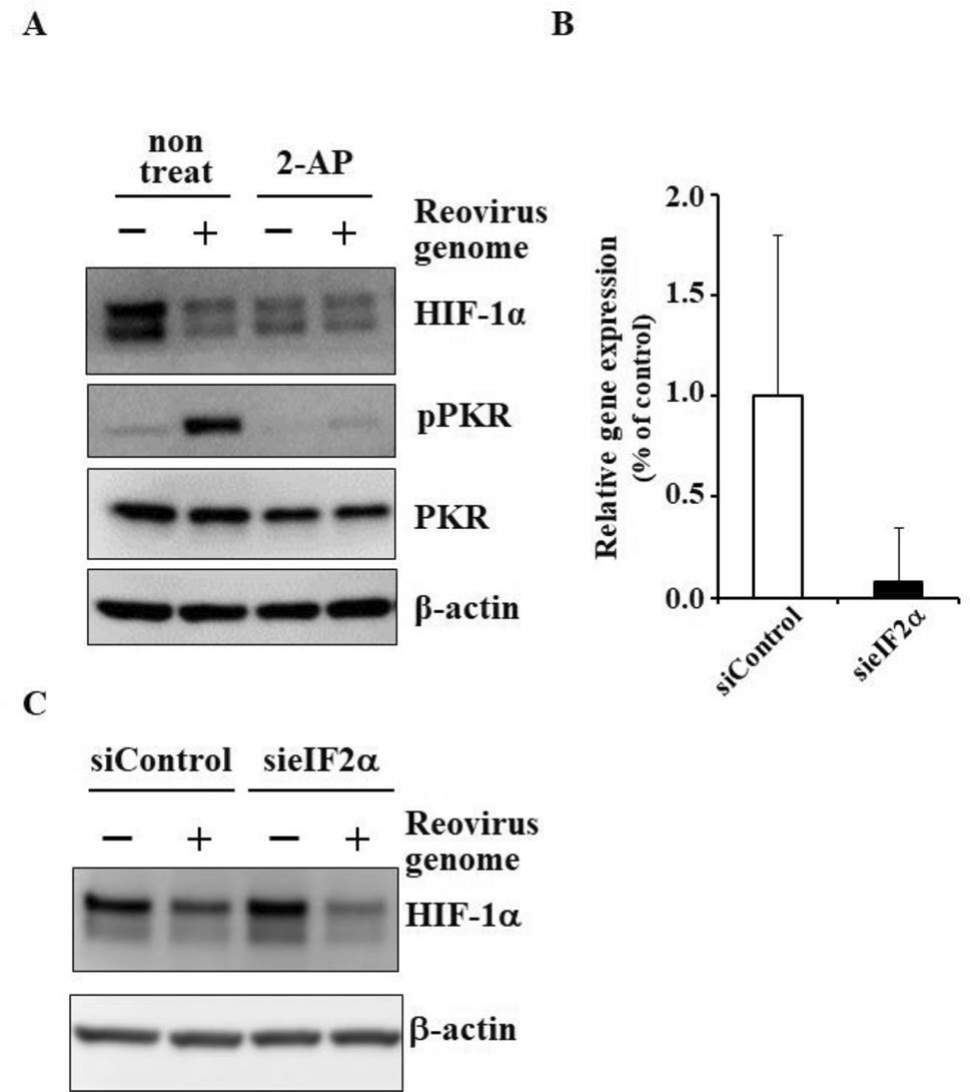
Kinase activity of PKR is necessary for dsRNA-mediated down-regulation of HIF-1α. **A** dsRNA-mediated down-regulation of HIF-1α in the presence of a PKR inhibitor, 2-AP. H1299 cells were pretreated with 2-AP at 10 mM for 2 h, followed by transfection with the reovirus dsRNA genome at 4 ng/ml for 24 h. **B** Knockdown efficiency of an siRNA against eIF2 in H1299 cells. H1299 cells were transfected with an siRNA against eIF2α at 50 nM for 24 h, followed by real-time RT-PCR analysis. **C** HIF-1α expression levels in eIF2α-knocked down cells following transfection with dsRNA. H1299 cells were transfected with an siRNA against eIF2α for 24 h, followed by transfection with the reovirus dsRNA genome at 4 ng/ml for 24 h. Data are expressed as means ± S.D. (n = 4). The representative images from at least two independent experiments are shown.

In order to examine whether phosphorylation activity of PKR is crucial for dsRNA-mediated reduction in HIF-1α protein levels, cells were pretreated with a PKR inhibitor, 2-AP. Phosphorylated PKR levels were largely reduced in 2-AP-pretreated cells following dsRNA transfection, indicating that 2-AP efficiently inhibited PKR (Fig. 2A). Pretreatment with 2-AP apparently canceled the dsRNA-mediated down-regulation of HIF-1α, although 2-AP alone significantly reduced HIF-1α protein levels in mock-transfected cells. These data suggested that the phosphorylation activity of PKR was crucial for dsRNA-mediated reduction in HIF-1α protein levels.

Next, in order to examine whether activated PKR-mediated inhibition of eIF2α was involved in dsRNA-mediated down-regulation of HIF-1α protein levels, eIF2α was knocked down by an siRNA. It is well known that eIF2α is phosphorylated by activation of PKR, resulting in suppression of translation [19]. More than 80% knockdown of eIF2α mRNA levels was found following transfection with an siRNA (Fig. 2B). HIF-1α protein levels were not restored by eIF2α knockdown in dsRNA-transfected cells (Fig. 2C). These data indicated that activated PKR-mediated inhibition of eIF2α was not involved in dsRNA-induced down-regulation of HIF-1α.

## Ubiquitination of HIF-1α was Induced Following Transfection with dsRNA

**Fig. 3:**
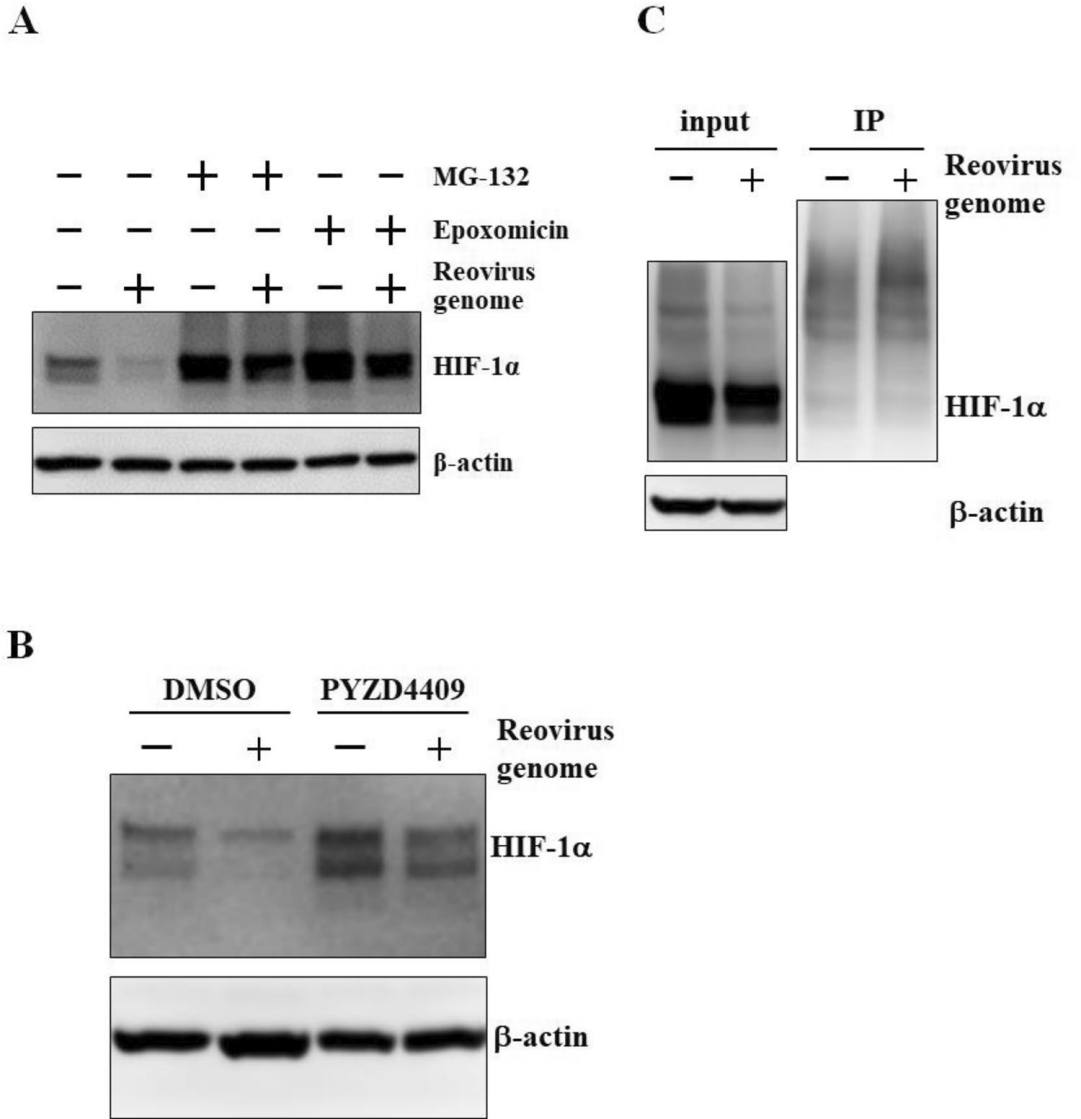
Induction of proteasomal degradation of HIF-1α in dsRNA-transfected cells. **A** Restoration of HIF-1α protein levels in dsRNA-transfected cells by pre-treatment with proteasome inhibitors. H1299 cells were pretreated with MG-132 and epoxomicin for 30 min, followed by transfection with the reovirus dsRNA genome at 4 ng/ml for 24 h. **B** Ubiquitination levels of HIF-1α following transfection with dsRNA. H1299 cells were transfected with the reovirus dsRNA genome at 4 ng/ml for 24 h. Ubiquitinated proteins were immune-precipitated, followed by western blotting analysis using anti-HIF-1 antibody. The representative images from at least two independent experiments are shown.

In order to examine whether the ubiquitin–proteasome system was involved in dsRNA-mediated down-regulation of HIF-1α, cells were pretreated with proteasome inhibitors, MG-132 and epoxomicin, followed by transfection with the reovirus dsRNA genome. A previous study reported that the ubiquitin–proteasome system was involved in reovirus-induced down-regulation of HIF-1α [13]. HIF-1α protein levels were significantly restored by pretreatment with the proteasome inhibitors in dsRNA-transfected cells (Fig. 3A). In addition, a specific inhibitor of the ubiquitin-activating enzyme UBA1, PYZD4409, significantly restored the HIF-1α protein levels in dsRNA-transfected cells (Fig. 3B). These data suggested that the ubiquitin–proteasome system is crucial for dsRNA-induced down-regulation of HIF-1α.

Next, in order to examine whether ubiquitination of HIF-1α was enhanced by transfection with dsRNA, ubiquitination levels of HIF-1 were evaluated by western blotting analysis. Ubiquitination levels of HIF-1α were promoted following transfection with the reovirus dsRNA genome (Fig. 3C). These results indicated that degradation of HIF-1α via the ubiquitin–proteasome system was promoted following transfection with dsRNA. However, dsRNA-induced down-regulation of HIF-1α was not canceled in the cells when VHL and RACK1, which play a crucial role in ubiquitin–proteasome-dependent degradation of HIF-1α [6, 8], were knocked down with an siRNA (Supplemental Figs. 4, 5). These data indicated that other molecules different from VHL or RACK1 were involved in dsRNA-induced degradation of HIF-1α.

## dsRNA-Induced Proteasomal Degradation of Other Proteins

**Fig. 4:**
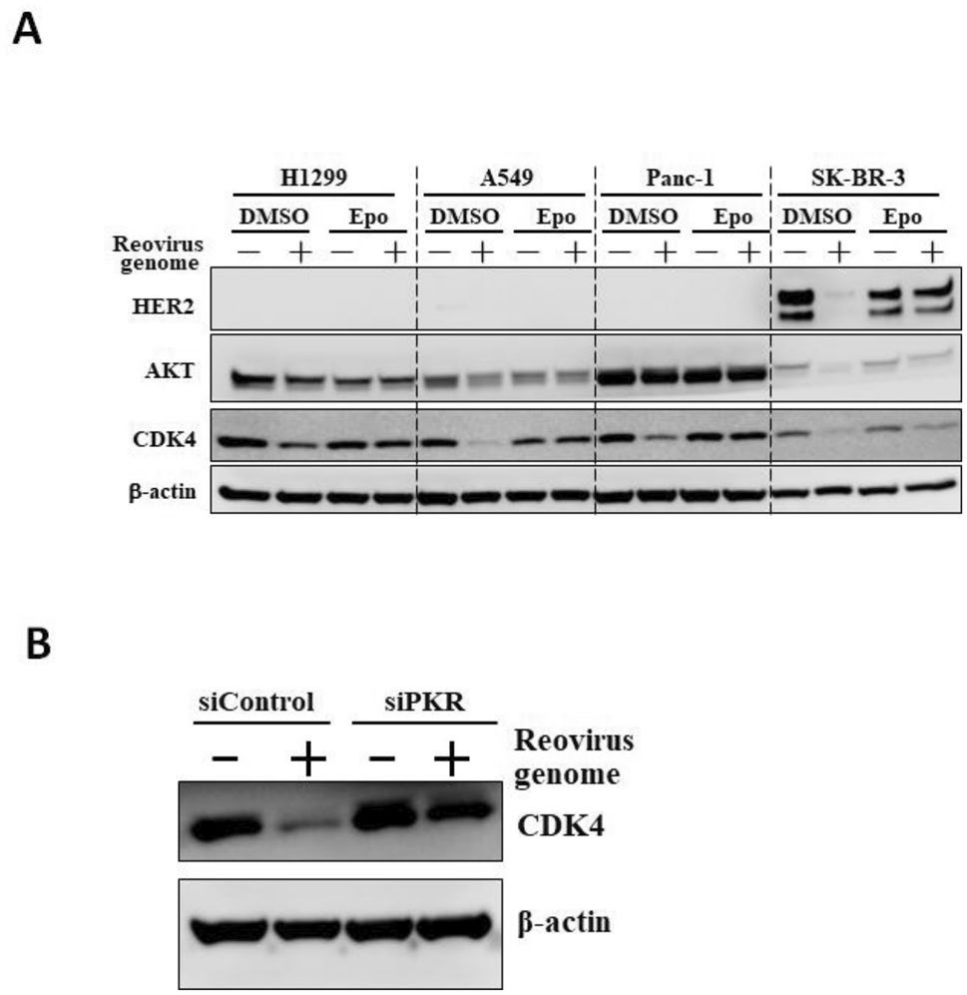
Induction of proteasomal degradation of CDK4, AKT, and HER2 following transfection with dsRNA. **A** Expression levels of CDK4, AKT, and HER2 in human tumor cells following transfection with dsRNA. Cells were pretreated with DMSO or epoxomicin for 30 min, followed by transfection with the reovirus dsRNA genome at 4 ng/ml for 24 h. DMSO, dimethyl sulfoxide; Epo, Epoxomicin. **B** Restoration of CDK4 expression by knockdown of PKR in dsRNA-transfected cells. H1299 cells were transfected with an siRNA against PKR for 24 h, followed by transfection with the reovirus dsRNA genome at 4 ng/ml for 24 h. The representative images from at least two independent experiments are shown.

In order to examine whether transfection with dsRNA induced a reduction in the expression of other proteins, western blotting analyses of several proteins were carried out following transfection with the reovirus dsRNA genome. Among the proteins examined, CDK4 protein levels were efficiently down-regulated by transfection with dsRNA in all the cells examined (Fig. 4A). AKT expression was also reduced in all dsRNA-transfected cells except for Panc-1 cells. Transfection with dsRNA also resulted in an apparent reduction in HER2 protein levels in SK-BR3 cells. dsRNA-mediated reduction in the expression of these proteins was significantly restored by a proteasome inhibitor, epoxomicin. Furthermore, knockdown of PKR apparently canceled the dsRNA-induced down-regulation of CDK4 (Fig. 4B). These data suggested that transfection with dsRNA resulted not only in a reduction of HIF-1α protein levels but also a reduction of other proteins in a PKR- and proteasome-dependent manner.

## Discussion

In this study, we found that PKR was involved in proteasomal degradation of HIF-1α protein levels following transfection with dsRNA in cultured cells. To the best of our knowledge, this is the first study demonstrating that PKR is involved in the induction of proteasomal degradation of proteins. PKR is a pattern recognition receptor recognizing dsRNAs of more than 33-bp length [20]. Following binding of dsRNA to PKR, PKR is dimerized and autophosphorylated. PKR phosphorylates eIF2α, leading to suppression of protein synthesis. In this study, phosphorylation of PKR was induced following dsRNA transfection, but knockdown of eIF2α did not cancel the dsRNA-induced reduction in the HIF-1α protein levels, indicating that PKR-mediated phosphorylation of eIF2α was not involved in the dsRNA-induced reduction in HIF-1α protein levels.

HIF-1α has been shown to be involved in various diseases and conditions, including inflammation [21, 22]. Furthermore, recent studies reported that HIF-1α promoted infection with various viruses, including severe acute respiratory syndrome coronavirus 2 (SARS-CoV-2) and respiratory syncytial virus [23, 24]. Infection with several viruses up-regulated HIF-1α protein levels because several virus proteins inhibited degradation of HIF-1α [25, 26]. These findings from previous studies and our present experiments suggest that PKR would function as an antivirus system by not only phosphorylating eIF2α but also promoting degradation of HIF-1α following recognition of dsRNA.

Recently, relationship between type I interferons (IFNs) and HIF-1α has been reported [27–29]. An increase in RIG-I mRNA levels and release of IFN- in the culture supernatants were observed under hypoxic conditions; on the other hand, type I IFNs did not stabilize HIF-1α [29]. Expression of type I IFNs is significantly induced following transfection with dsRNA. RIG-I and MDA5 play a major role in dsRNA-mediated production of various types of cytokines, including type I IFNs. Type I IFN expression was largely reduced in IPS-1-knocked down cells following infection with reovirus [30]. However, this study demonstrated that neither RIG-I nor MDA5 was involved in dsRNA-induced down-regulation of HIF-1α, suggesting that dsRNA-induced expression of type I IFNs did not largely affect HIF-1 protein levels.

This study demonstrated that expression levels of not only HIF-1α but also the other proteins, including CDK4 and AKT, were reduced in dsRNA-transfected cells. A proteasome inhibitor, epoxomicin, restored the expression levels of these molecules in dsRNA-transfected cells, suggesting that proteasomal degradation of these proteins was induced by dsRNA transfection. CDK4, AKT, and HER2 are involved in cell proliferation, indicating that these molecules would be promising targets for cancer therapy [29, 31, 32]. dsRNA-mediated down-regulation of CDK4, AKT, and HER2 resulted in the suppression of tumor cell proliferation. Previous studies reported that dsRNA suppressed cell proliferation and induced cell death after transfection in cultured cells when dsRNA doses higher than those used in this study were added to the cells [33, 34]. dsRNA-mediated down-regulation of CDK4, AKT, and HER2 at least partly contributed to dsRNA-mediated suppression of cell growth and cytotoxicity.

Several groups, including ours, previously reported that reovirus induced down-regulation in HIF-1α protein levels in cultured cells [10, 11, 13, 35]. However, reovirus-induced reduction in HIF-1α protein levels was found in PKR-knockdown cells, indicating that the reovirus-induced reduction in HIF-1α protein levels occurred in a PKR-independent manner. Previous studies reported that reovirus protein sigma3 inhibited PKR, indicating that PKR was inhibited by sigma3 in reovirus-infected cells [36, 37]. Furthermore, Bussiere et al. reported that a dsRNA genome-deficient virion significantly suppressed HIF-1α expression at a level similar to the infectious virion [38]. Therefore, other mechanisms not involving the PKR-dependent pathway must have been involved in reovirus-mediated down-regulation of HIF-1α.

In this study, reovirus double-stranded RNA genome was used as a model of virus-derived double-stranded RNA. It is well known that PKR recognizes double-stranded RNA, however, several studies demonstrated that stem-loop-containing single-stranded RNA also mediated PKR activation [39, 40]. Several single-stranded RNA virus genomes, including influenza virus genome and hepatitis virus genome, contains complicated secondary structure. Such single-stranded RNA virus genomes might induce proteasomal degradation of HIF-1α via a PKR-dependent manner, similarly to reovirus dsRNA genome.

In summary, we demonstrated that dsRNA induced proteasomal degradation of not only HIF-1α but also several proteins in cultured cells in a PKR-dependent manner. This study provides important information not only for therapeutic applications of dsRNA but also for elucidating the mechanism of regulation of HIF-1α expression levels. The mechanism by which dsRNA-mediated activation of PKR promotes proteasomal degradation will be the focus of future studies.

### Supplementary Information

Below is the link to the electronic supplementary material.

Supplementary file1 (PDF 1071 KB)

Supplementary file2 (PDF 86 KB)

## Data Availability

The datasets analyzed during this study are available from the corresponding author on reasonable request.
