# Supplementary material for: Involvement of Protein Kinase R in Double-Stranded RNA-Induced Proteasomal Degradation of Hypoxia Inducible Factor-1α
Source: Inflammation. 2023 Aug 24;46(6):2332–42. doi: 10.1007/s10753-023-01881-8 (PMC10673737; doi:10.1007/s10753-023-01881-8)
Supplement: Supplementary file 1 — Supplementary file1 (PDF 1071 KB) [file 10753_2023_1881_MOESM1_ESM.pdf]

Supplemental Fig.1

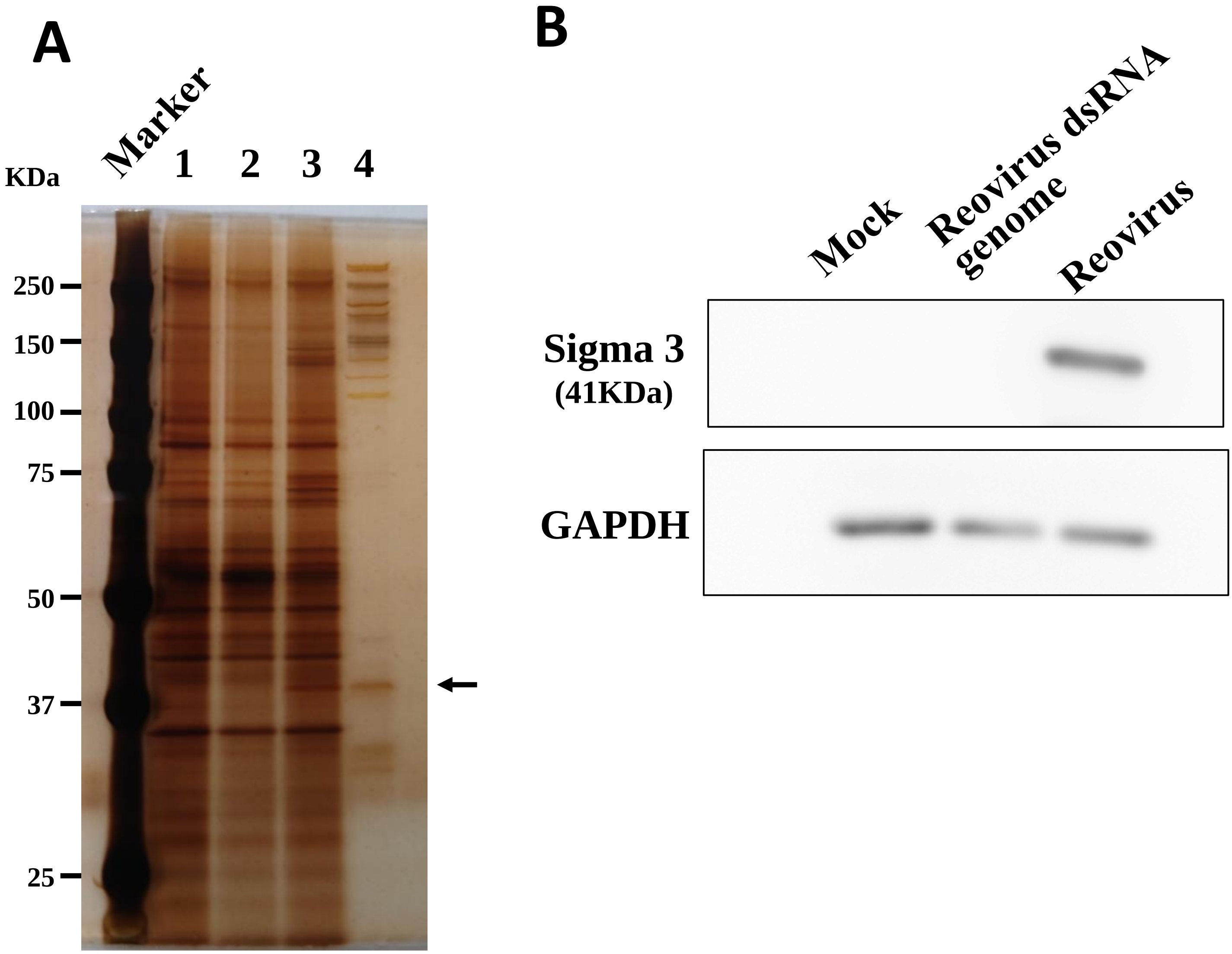

1. **Mock**
2. **Reovirus dsRNA genome**
3. **Reovirus**
4. **Purified reovirus**

Supplemental Fig.1
Undetectable levels of virus protein expression following transfection with the reovirus dsRNA genome. H1299 cells were seeded on a 12-well plate at a density of $5x10^4$ cells/well. On the following day, cells were transfected with the reovirus dsRNA genome at 4 ng/mL using Lipofectamine RNAiMAX, and were infected with reovirus at a multiplicity of infection (MOI) of 50. Following a 24-h incubation, cells were recovered, followed by SDS-PAGE and silver staining of cellular proteins (A) and western blotting analysis of sigma 3 protein expression in the cells (B). The arrow at the right side indicates the bands predicted for sigma 3 protein.

Supplemental Fig.2

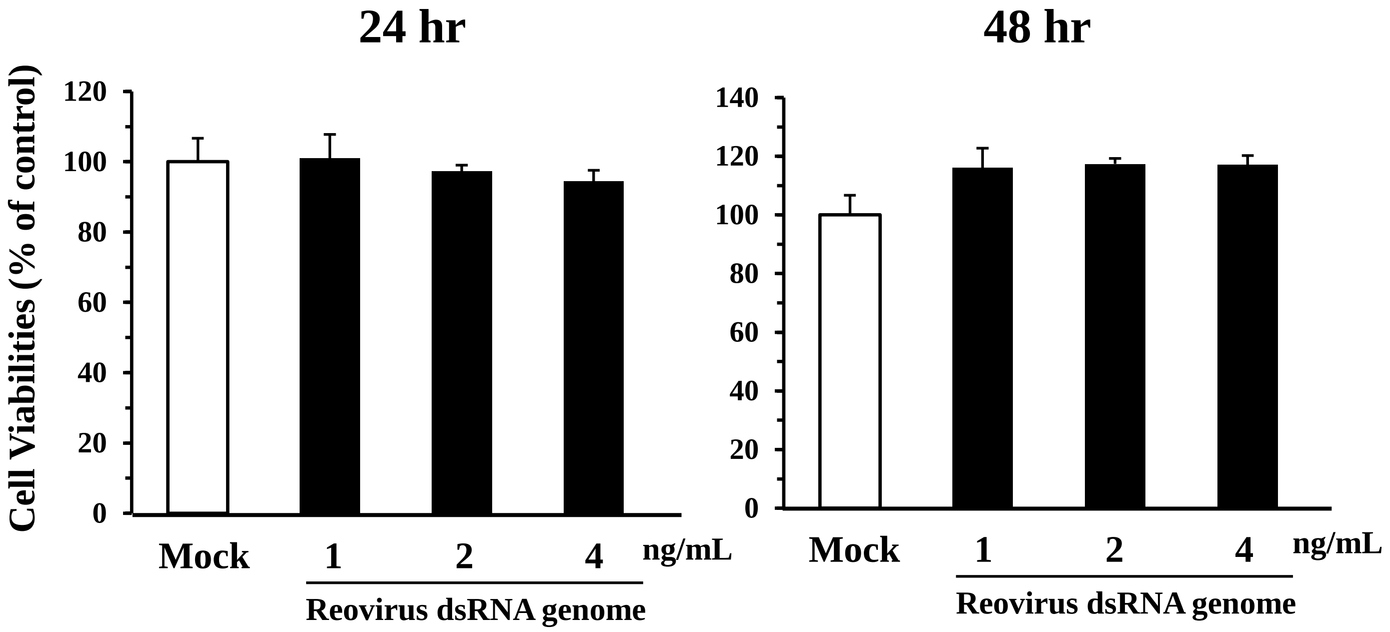

Supplemental Fig.2

Cell viabilities following transfection with the reovirus dsRNA genome. H1299 cells were seeded on a 96-well plate at a density of 1×10^4 cells/well. On the following day, cells were transfected with the reovirus dsRNA genome at the indicated doses using Lipofectamine RNAiMAX. Following 24-h and 48-h incubation, cell viabilities were determined by a WST-8 assay. Data are expressed as means ± S.D. (n=4).

Supplemental Fig.3

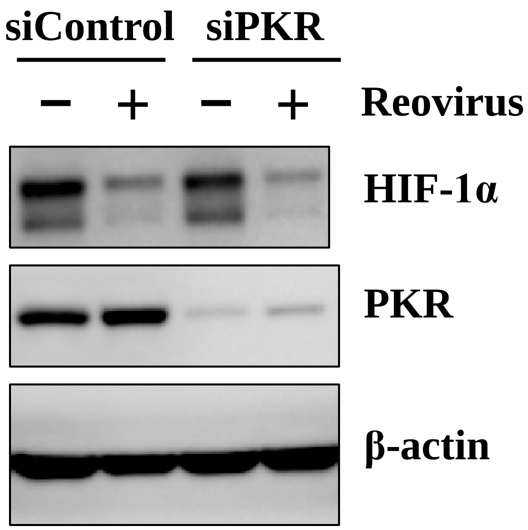

Supplemental Fig.3

Reovirus-mediated down-regulation of HIF-1α in a PKR-independent manner. H1299 cells were transfected with an siRNA against PKR at 50 nM. Following a 24-h incubation, cells were infected with reovirus at an MOI of 20. Cell lysates were prepared 24 h after infection, followed by western blotting analysis. Representative images of two independent experiments are shown.

Supplemental Fig.4

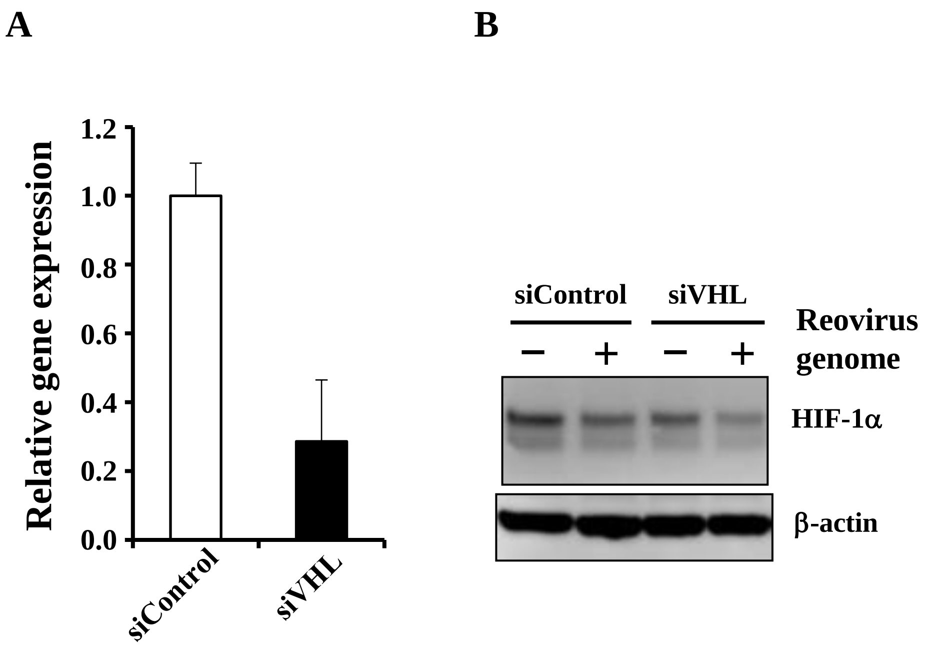

Supplemental Fig.4

dsRNA-mediated down-regulation of HIF-1α in a VHL-independent manner. H1299 cells were transfected with an siRNA against VHL at 50 nM. Following a 24-h incubation, cells were transfected with the reovirus dsRNA genome at 4 ng/mL. Real-time RT-PCR analysis was performed 24 h after siRNA transfection. Cell lysates were prepared 24 h after transfection with reovirus dsRNA genome, followed by western blotting analysis. Data are expressed as means ± S.D. (n=4). The representative images from at least two independent experiments are shown.

Supplemental Fig.5

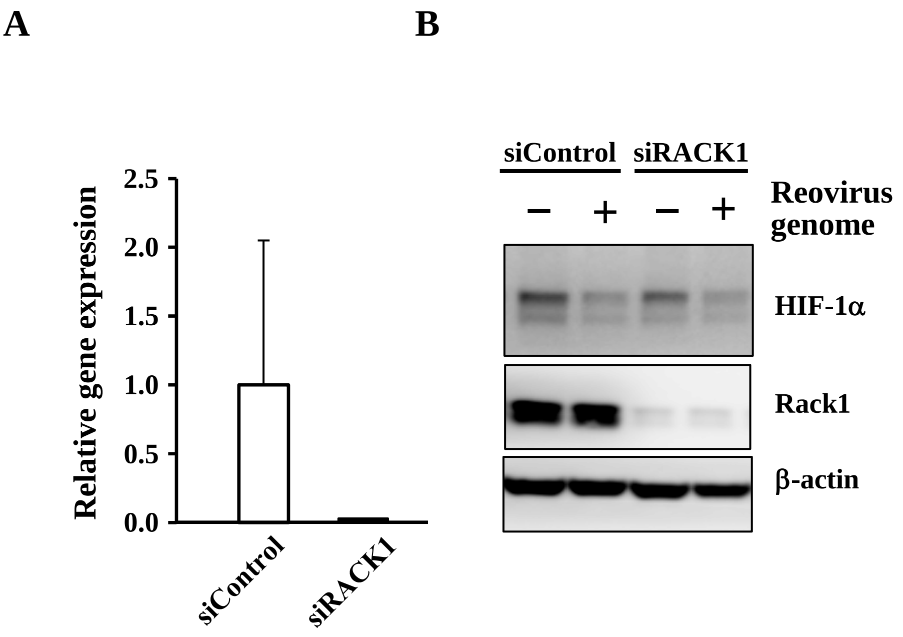

Supplemental Fig.4

dsRNA-mediated down-regulation of HIF-1α in a RACK1-independent manner. H1299 cells were transfected with an siRNA against RACK1 at 50 nM. Following a 24-h incubation, cells were transfected with the reovirus dsRNA genome at 4 ng/mL. Real-time RT-PCR analysis was performed 24 h after siRNA transfection. Cell lysates were prepared 24 h after transfection with the reovirus dsRNA genome, followed by western blotting analysis. Data are expressed as means ± S.D. (n=4). The representative images from at least two independent experiments are shown.

Supplemental Fig.6

HIF-1α

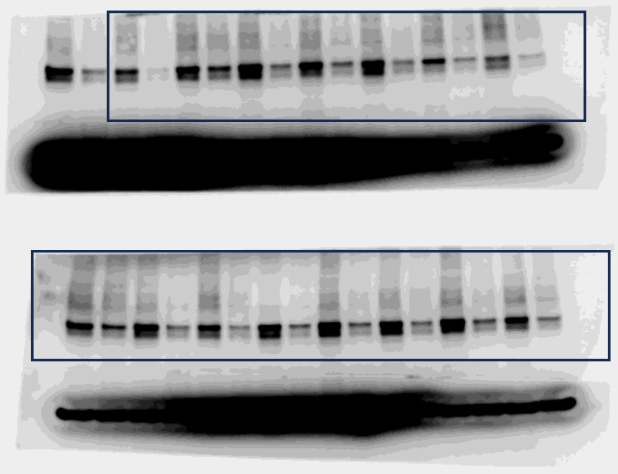

β-actin

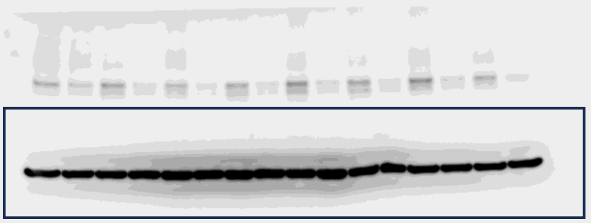

β-actin

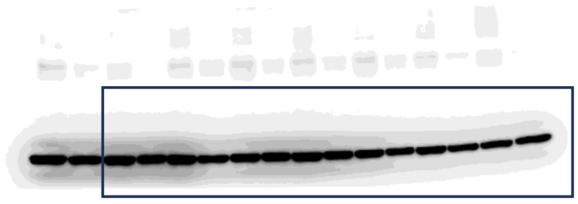

Supplemental Fig.6
Raw image of western blots for HIF-1α and β-actin in H1299 cells used in Figure 1B.

Supplemental Fig.7

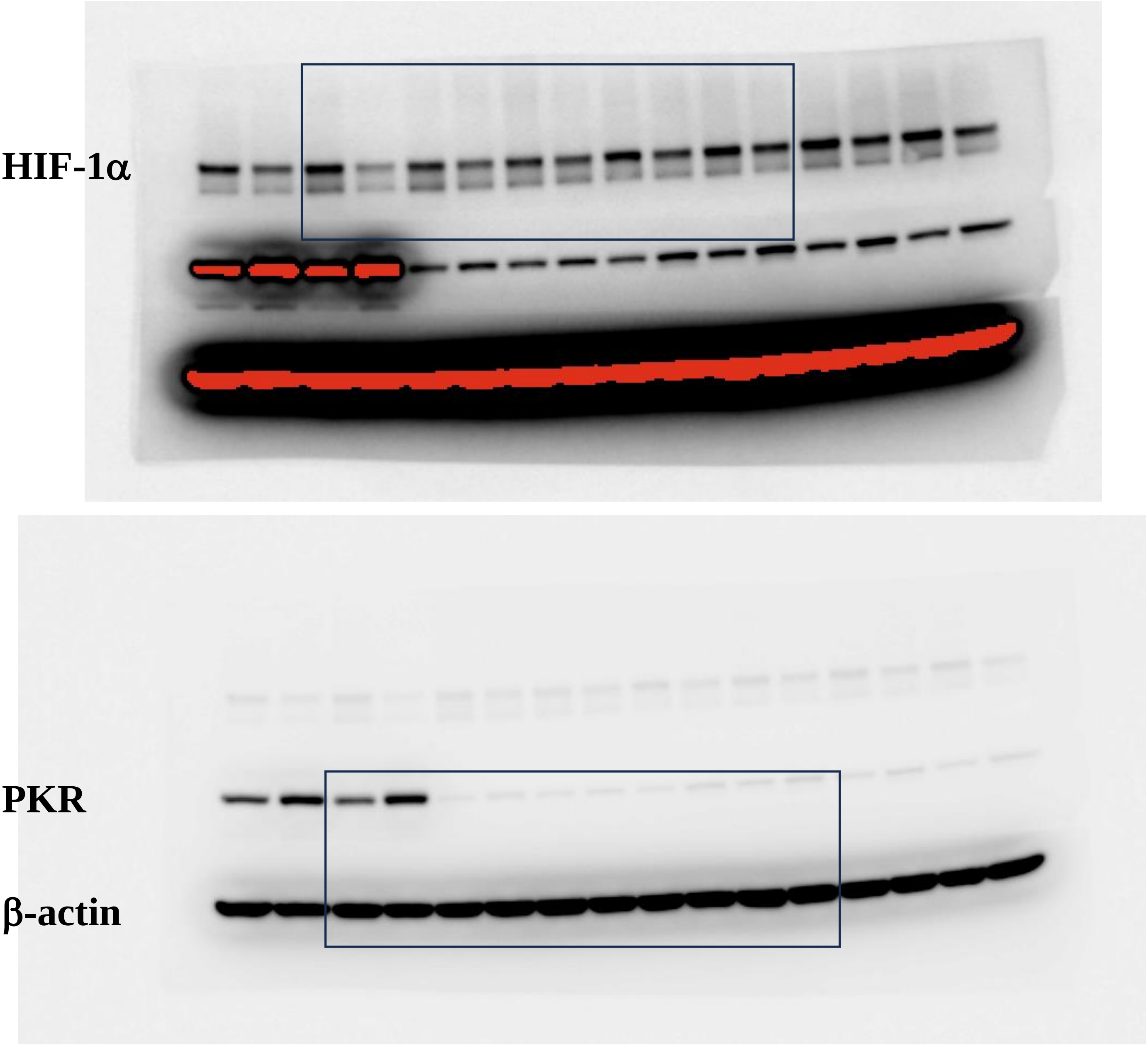

Supplemental Fig.7
Raw image of western blots for HIF-1α, PKR, and β-actin in H1299 cells used in Figure 1E.

Supplemental Fig.8

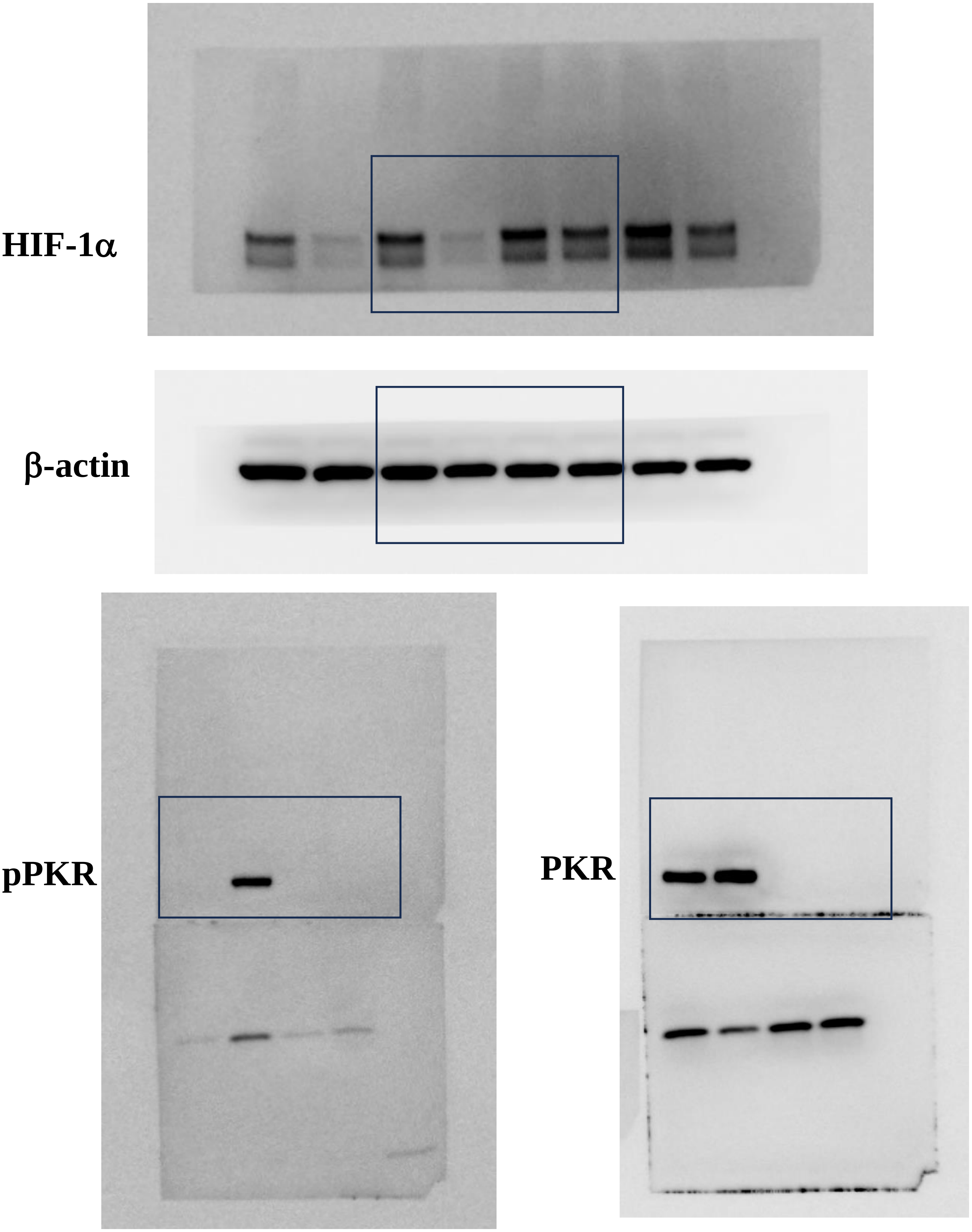

Supplemental Fig.8
Raw image of western blots for HIF-1α, PKR, phosphorylated PKR (pPKR), and β-actin in H1299 cells used in Figure 1F.

Supplemental Fig.9

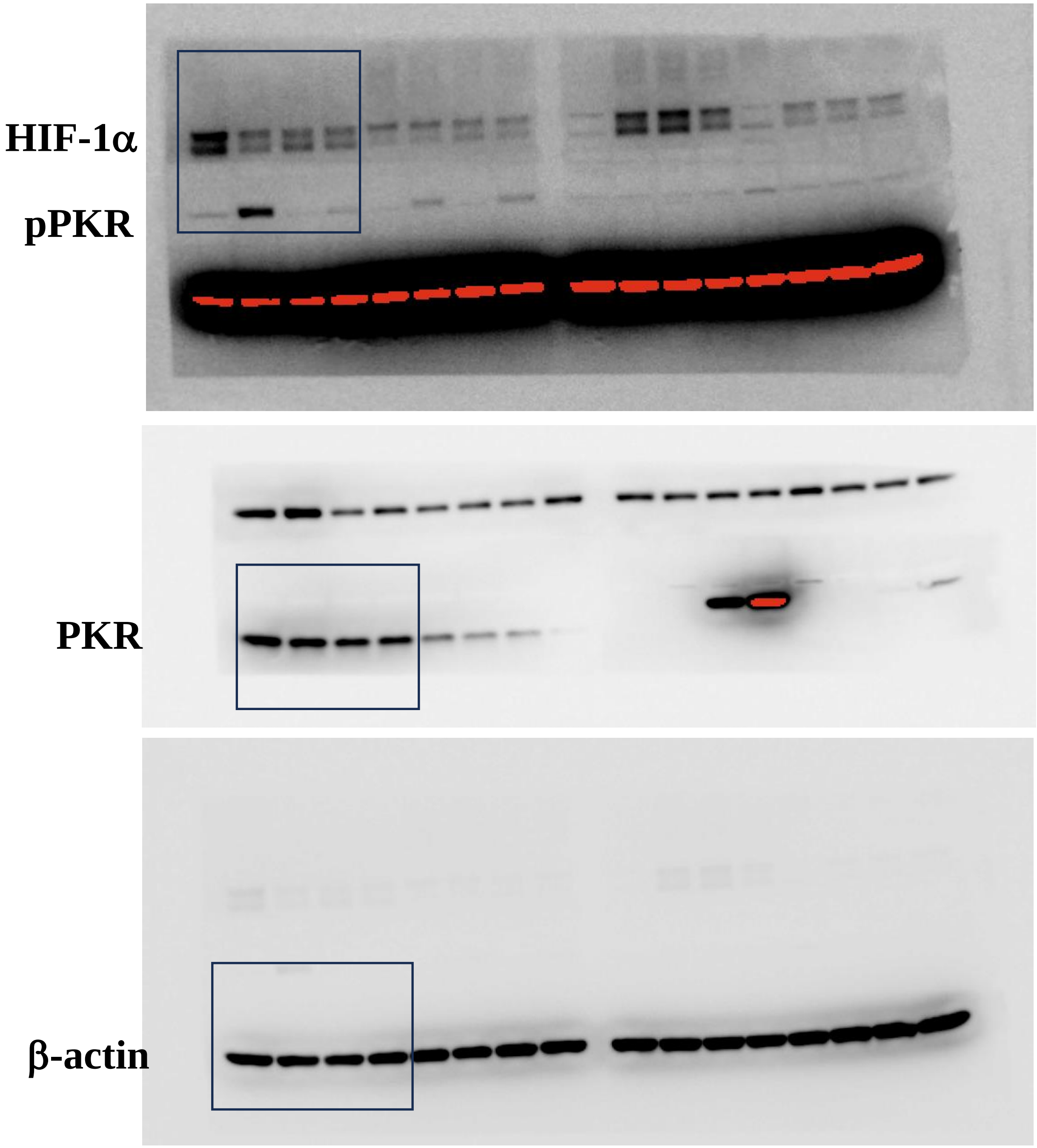

Supplemental Fig.9
Raw image of western blots for HIF-1α, PKR, phosphorylated PKR (pPKR), and β-actin in H1299 cells used in Figure 2A.

Supplemental Fig.10

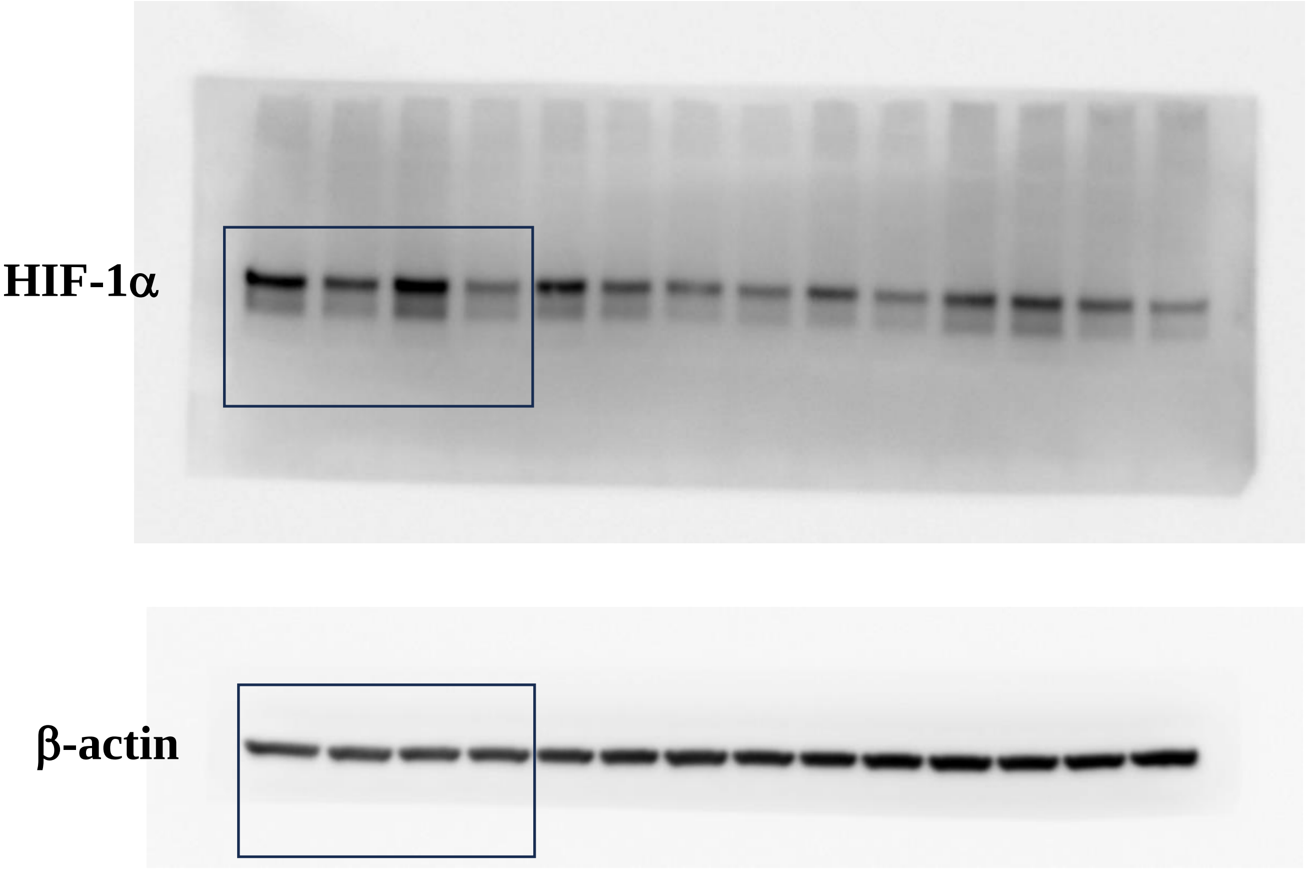

Supplemental Fig.10
Raw image of western blots for HIF-1α and β-actin in H1299 cells used in Figure 2C.

Supplemental Fig.11

HIF-1α

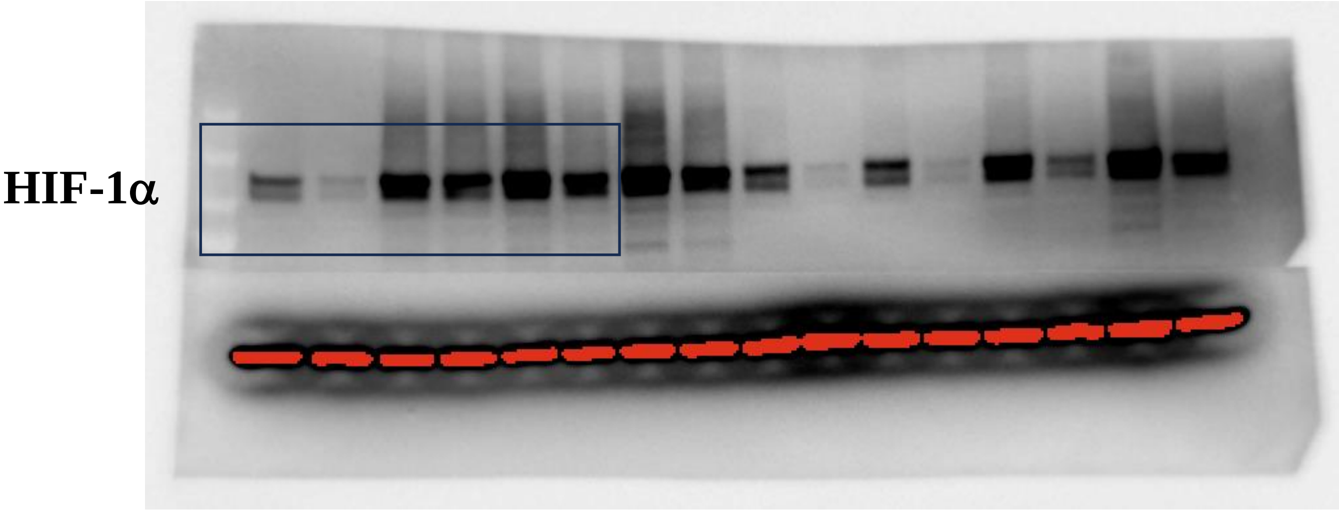

β-actin

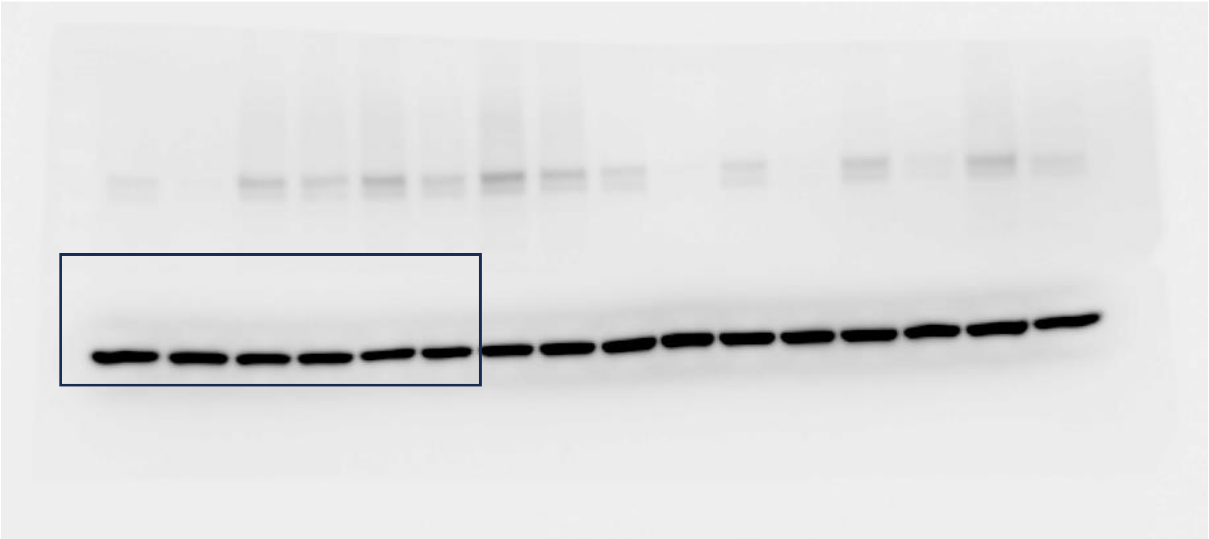

Supplemental Fig.11
Raw image of western blots for HIF-1α and β-actin in H1299 cells used in Figure 3A.

Supplemental Fig.12

HIF-1α

β-actin

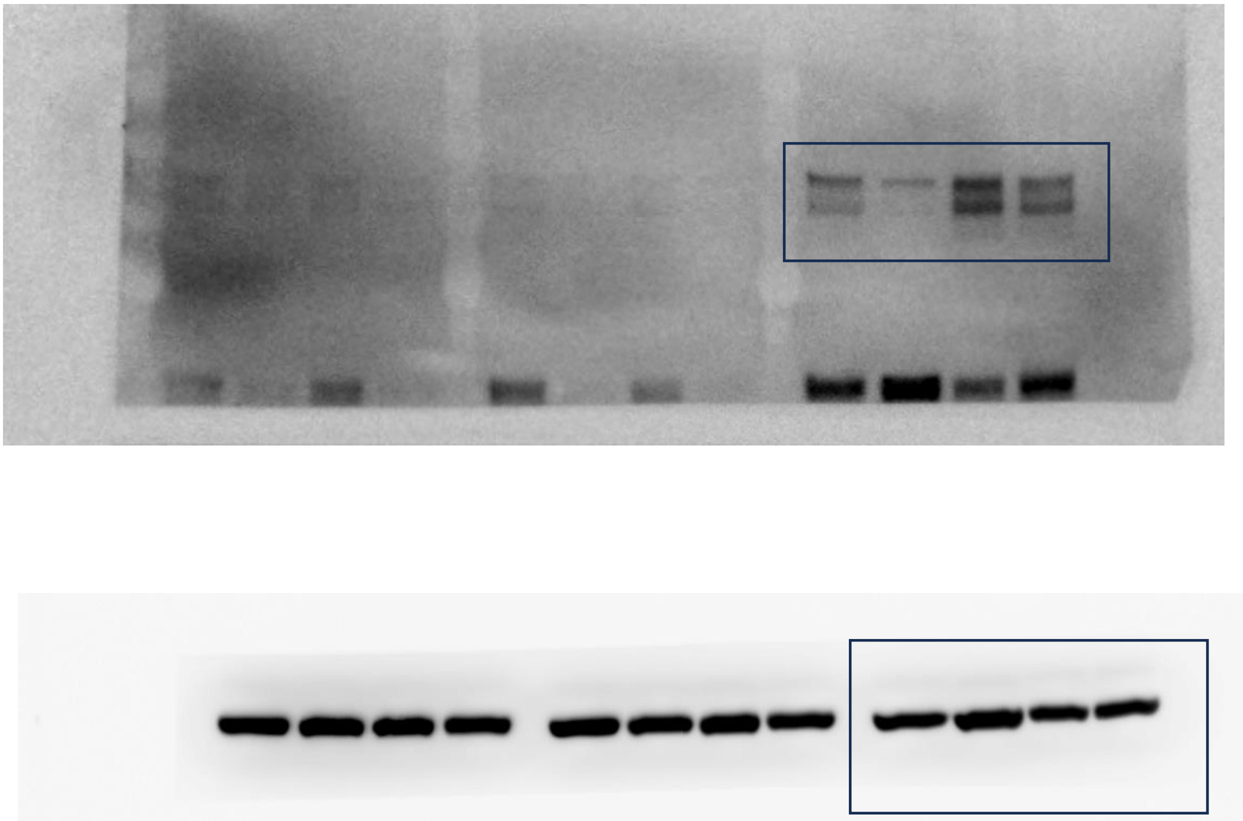

Supplemental Fig.12
Raw image of western blots for HIF-1α and β-actin in H1299 cells used in Figure 3B.

Supplemental Fig.13

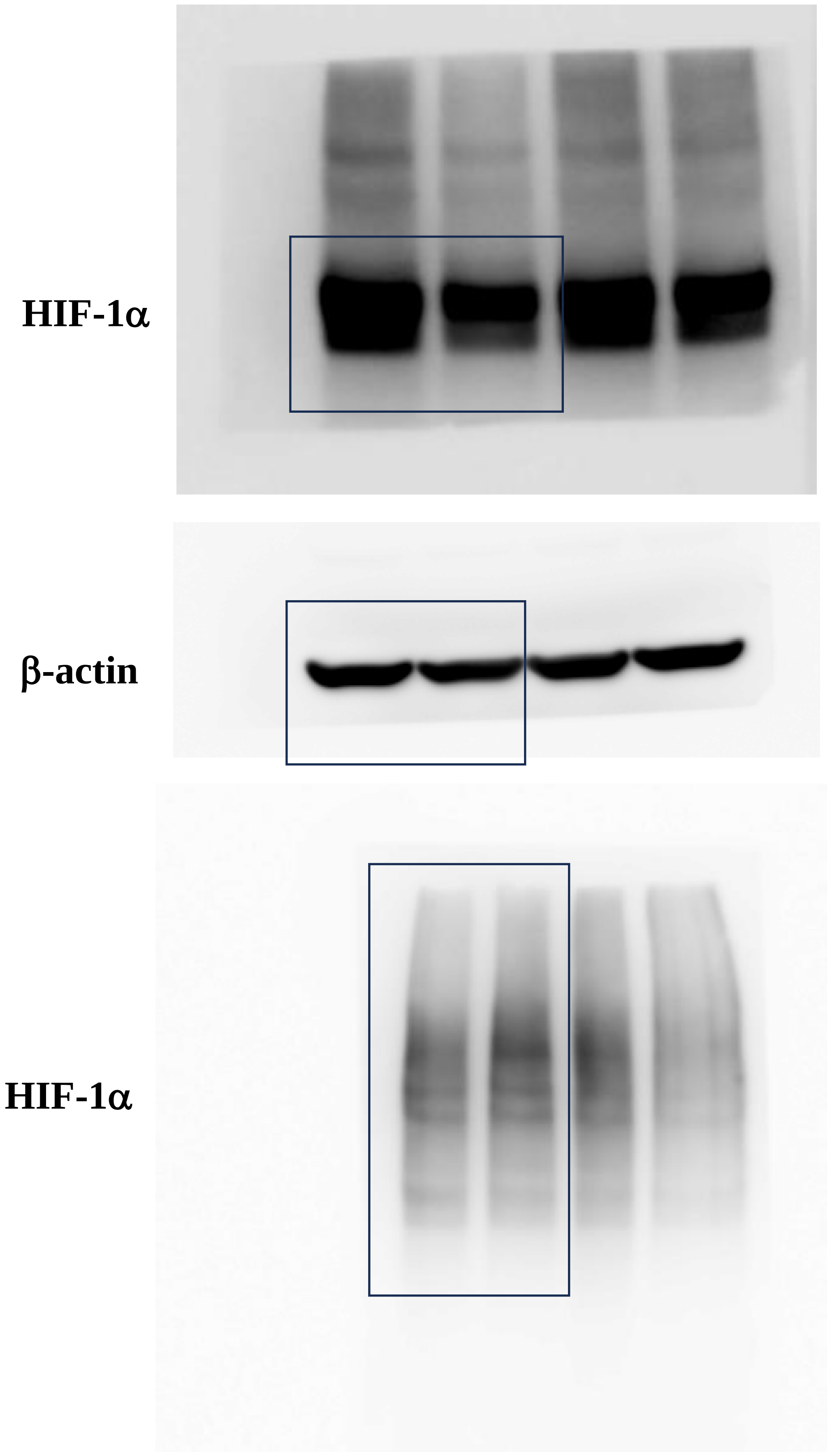

Supplemental Fig.13
Raw image of western blots for HIF-1α and β-actin in H1299 cells used in Figure 3C.

Supplemental Fig.14

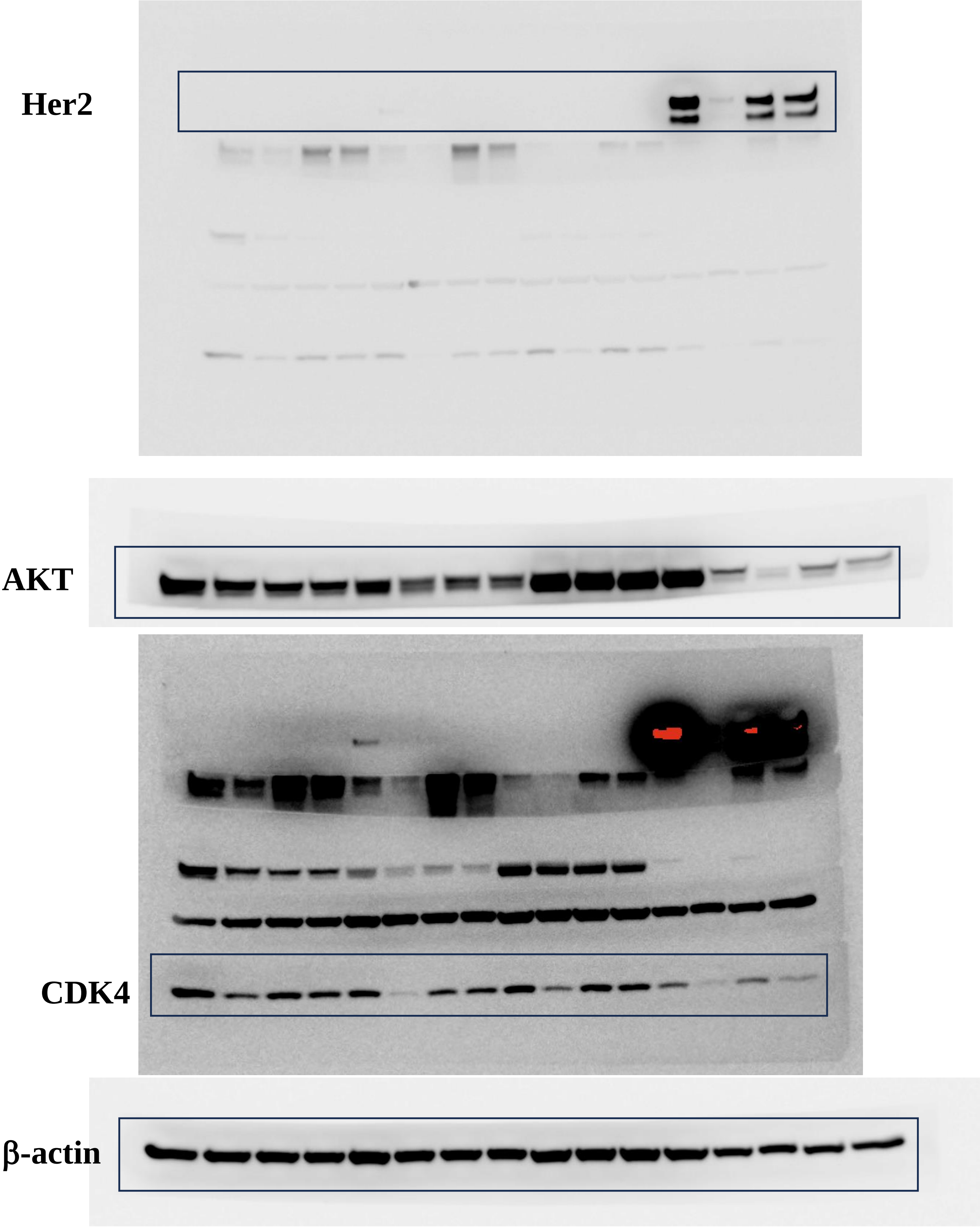

Supplemental Fig.14
Raw image of western blots for HIF-1α and β-actin in human cultured cells used in Figure 4A.

Supplemental Fig.15

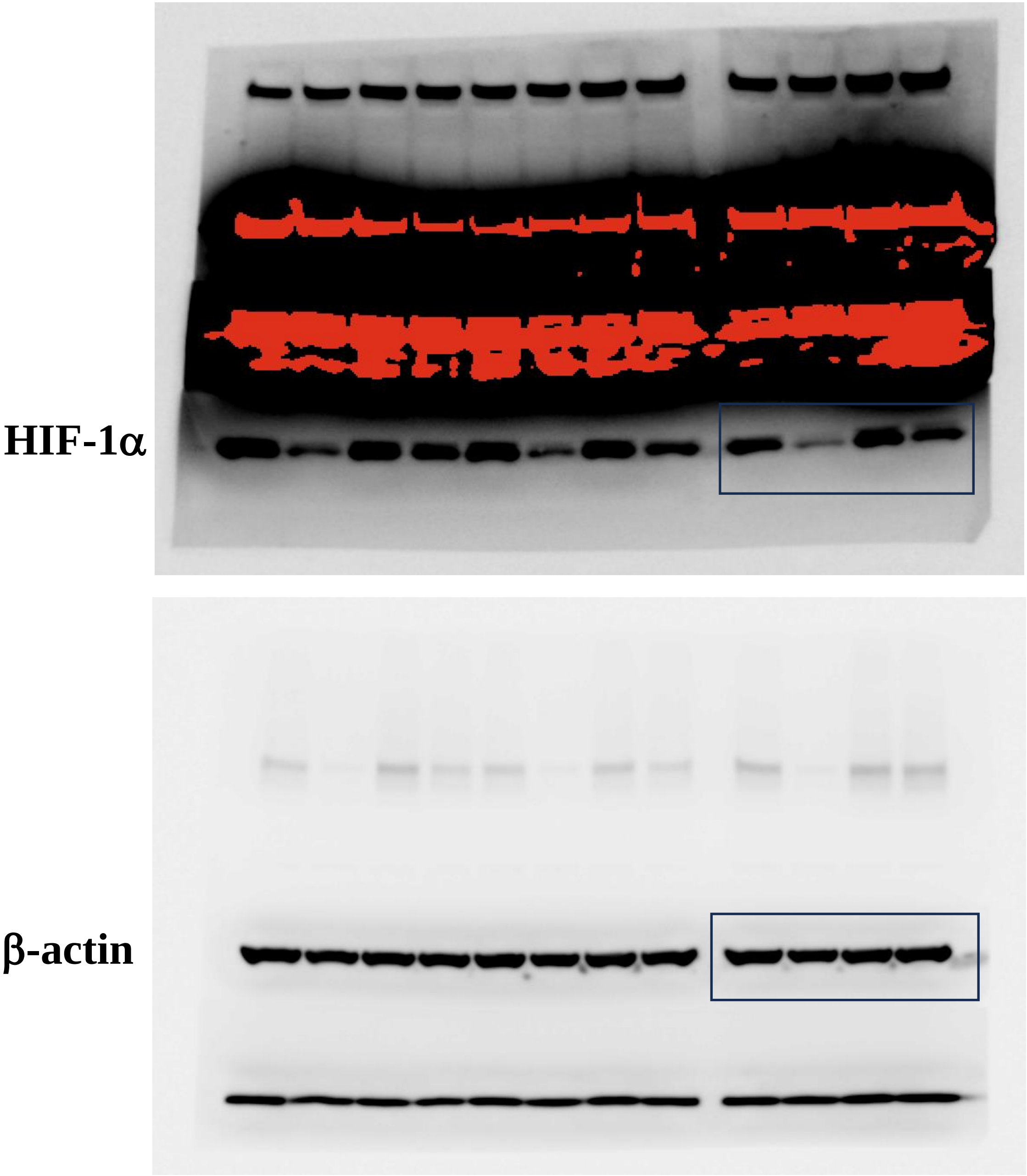

Supplemental Fig.15
Raw image of western blots for HIF-1α and β-actin in H1299 cells used in Figure 4B.

Supplemental Fig.16

Sigma 3

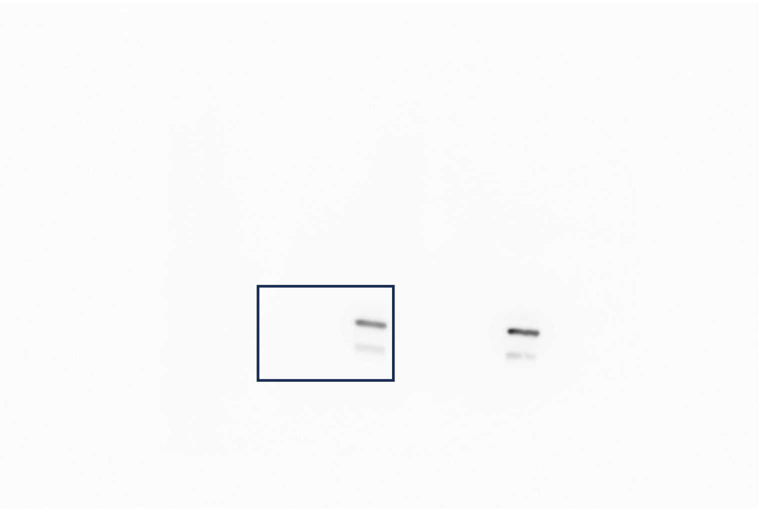

GAPDH

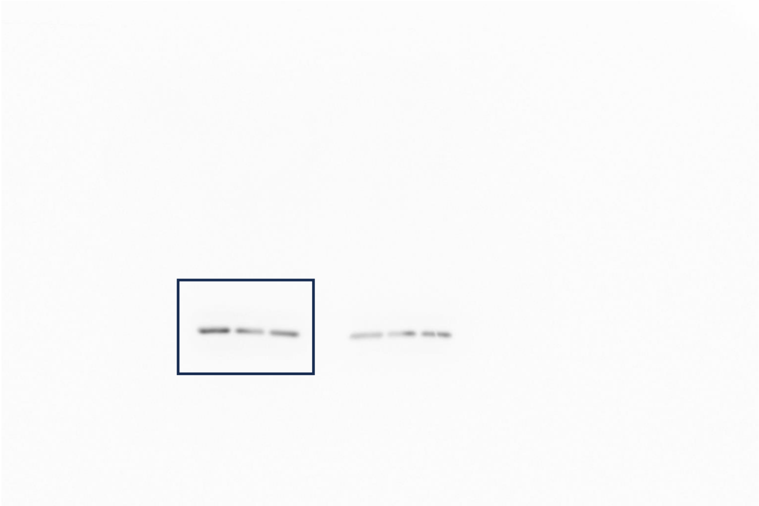

Supplemental Fig.16
Raw image of western blots for reovirus sigma 3 and GAPDH in H1299 cells used in Supplemental Figure 1B.

Supplemental Fig.17

HIF-1α

PKR

β-actin

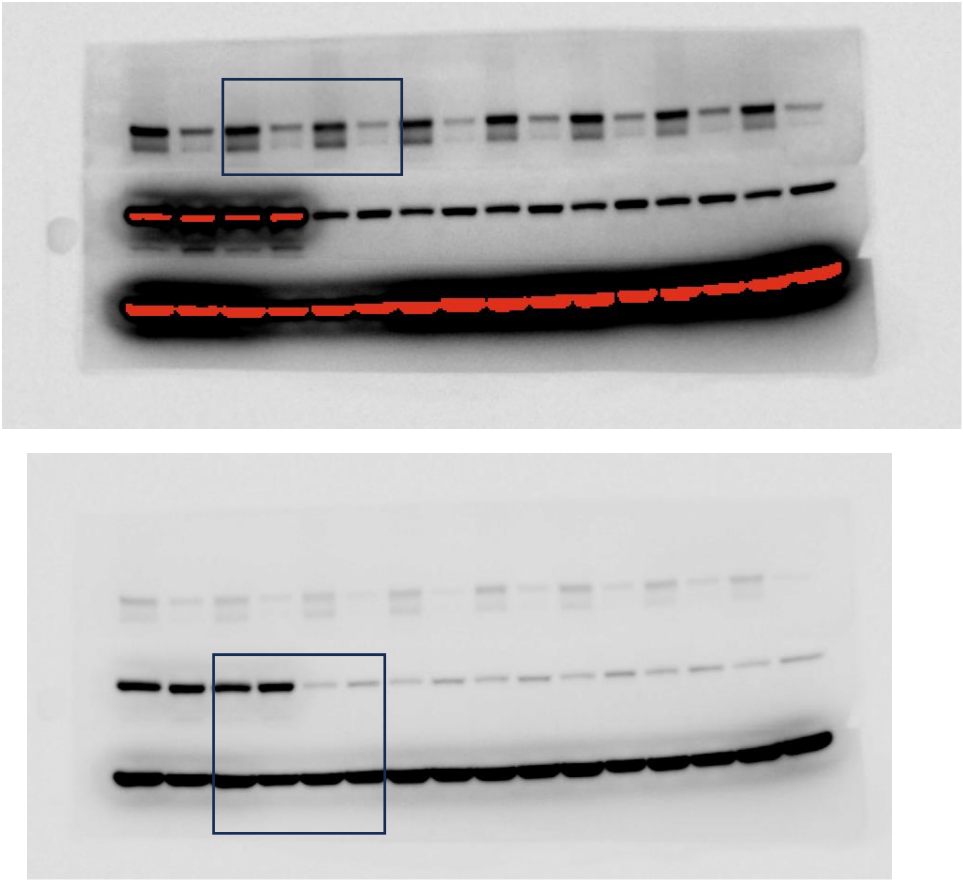

Supplemental Fig.17

Raw image of western blots for HIF-1α, PKR, and β-actin in H1299 cells used in Supplemental Figure 3.

Supplemental Fig.18

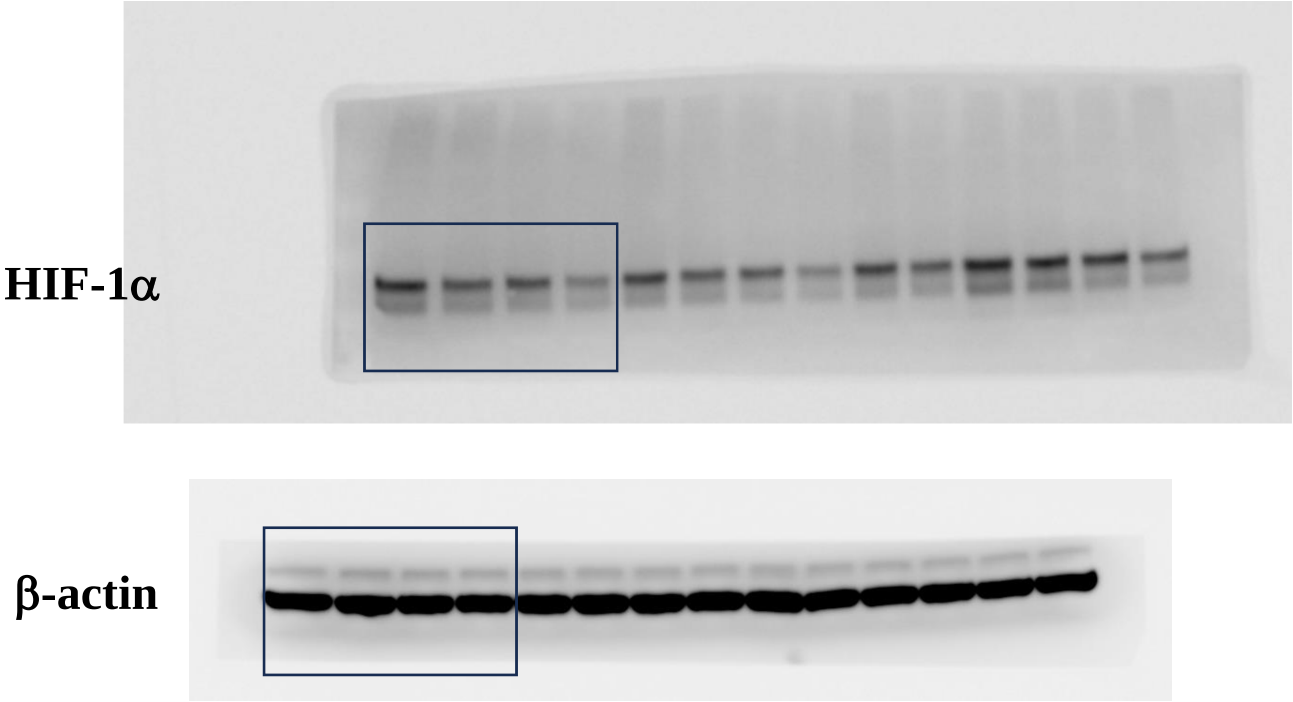

Supplemental Fig.18
Raw image of western blots for HIF-1α and β-actin in H1299 cells used in Supplemental Figure 4B.

Supplemental Fig.19

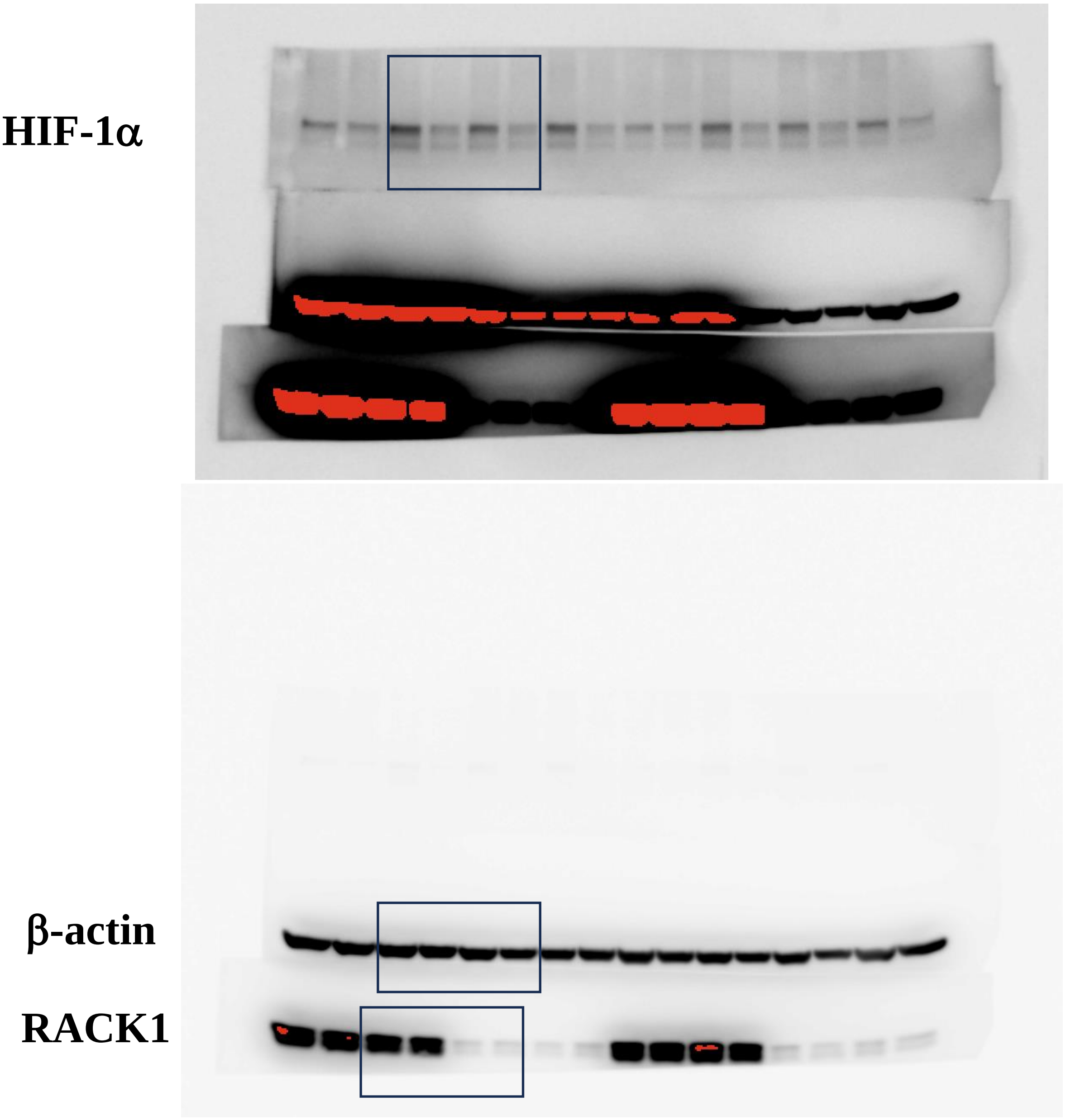

Supplemental Fig.19
Raw image of western blots for HIF-1α, RACK1, and β-actin in H1299 cells used in Supplemental Figure 5B.
