# Supplementary material for: Involvement of Protein Kinase R in Double-Stranded RNA-Induced Proteasomal Degradation of Hypoxia Inducible Factor-1α
Source: Inflammation. 2023 Aug 24;46(6):2332–42. doi: 10.1007/s10753-023-01881-8 (PMC10673737; doi:10.1007/s10753-023-01881-8)
Supplement: Supplementary file 2 — Supplementary file2 (PDF 86 KB) [file 10753_2023_1881_MOESM2_ESM.pdf]

**Supplemental Table 1.** Targeted sequences of siRNAs used in this study.

| siRNA | Targeted sequence (sense strand; 5' to 3') |
|---|---|
| siIPS-1 | UAGUUGAUCUCGCGGACGA |
| siTLR3 | CAGCAUCUGUCUUUAAUAA |
| siMyD88 | GGAGGAUUGCCAAAAGUAU |
| siTRIF | GACCAGACGCCACUCCAAC |
| siTBK1 | GACAGAAGUUGUGAUCACA |
| siDHX9 | GAAGUGCAAGCGACUCUAG |
| siPKR | GGUGAAGGUAGAUCAAAGA |
| siPKR-2 | GACGGAAAGACUUACGUUA |
